# Collegial Organizational Climate Alleviates Japanese Schoolteachers’ Risk for Burnout

**DOI:** 10.3389/fpsyg.2021.737125

**Published:** 2021-12-06

**Authors:** Hirofumi Hashimoto, Kaede Maeda

**Affiliations:** ^1^Graduate School of Literature and Human Sciences, Osaka City University, Osaka, Japan; ^2^Urban-Culture Research Center, Graduate School of Literature and Human Sciences, Osaka City University, Osaka, Japan; ^3^Japan Society for the Promotion of Science, Tokyo, Japan; ^4^Graduate School of Letters, Yasuda Women’s University, Hiroshima, Japan

**Keywords:** school psychology, burnout, collegial organizational climate in a school, psycho-educational services, help-seeking preference

## Abstract

The purpose of the current study was to examine the influence of individuals’ help-seeking preference (HSP) and their collective perception of the organizational climate in school on teachers’ mental health. Previous studies demonstrated that HSP was negatively associated with risk of burnout, suggesting that teachers who hesitate to seek help from their colleagues are more likely to have mental health problems. Thus, the current study hypothesized that a collegial organizational climate would be negatively associated with burnout. To test this hypothesis, we developed a scale to measure schoolteachers’ collective perception of their organizational climate (Study 1), and the newly developed scale was used to assess its relationship with HSP and teachers’ burnout risk (Study 2). The results demonstrated that younger teachers, a low level of help-seeking, and a less collaborative climate increased the risk of burnout. The results also showed a significant interaction effect, indicating that HSP was less closely associated with teachers’ burnout risk if their organization was perceived as having a collegial climate. These findings clearly show how the social environment of a school’s organizational climate can affect schoolteachers’ mental health in Japan.

## Introduction

Several problems need to be solved in Japanese school education, such as bullying, non-attendance, child abuse and neglect, and the necessity for enriching special needs education toward inclusion. Facing these difficult problems, Japanese schoolteachers are now struggling to cope with these issues. In recent years, Japanese schoolteachers have been required to have the ability to manage their classes and provide individual guidance and assistance to each student. It is very difficult for not only young or novice but also older or expert schoolteachers. In such a rapidly changing and challenging educational environment, many Japanese schoolteachers are devoted to teaching, guiding, and supporting students. However, it is not so hard to imagine that they will be exhausted or have mental exhaustion due to receiving criticism and complaints from their students, the students’ parents, and others, or receiving negative feedback from managers in an environment where schoolteachers cannot or do not support each other. In this sense, the current situation in which the educational environment surrounding Japanese schoolteachers can increase the risk of burnout is extremely problematic. This is because it deteriorates the mental health of schoolteachers, and as a result, leads to the lowering of psycho-educational services in Japan.

These concerns, as described above, are borne from data collected for the Teaching and Learning International Survey conducted by the Organization for Economic Co-operation and Development (OECD) in 2013 (Organization for Economic Co-Operation and Development, 2014). The survey demonstrated that of the 34 countries and regions surveyed, schoolteachers in Japan had the longest working hours. Compared with the average working time of 38.3 h per week, working hours for schoolteachers in Japan, around 53.9 h per week, were much longer than their peers in other countries. A similar survey published about 4 years later (Organization for Economic Co-operation and Development, 2019) also confirmed the prominence of long working hours among teachers in Japan. Additionally, the mental health issue among Japanese schoolteachers has become a pressing issue in recent years that needs to be solved with some urgency. According to a survey conducted by the Ministry of Education, Culture, Sports, Science and Technology (MEXT), although the number has been gradually decreasing after peaking at 5,458 in 2009, the number of leaves of absence due to mental health problems has become more pronounced, and over the past decade, nearly 5,000 schoolteachers per year have taken sick leave for mental health problems (Ministry of Education, Culture, Sports, Science and Technology, 2020). These two data do not seem to be directly related; however, it is reasonable to interpret that Japanese schoolteachers’ long working hours have the potential to cause mental health problems. If so, what could be important factors that can be useful in considering these mental health issues, or specifically, to reduce the schoolteachers’ risk of burnout in such a challenging environment?

While there have been a bunch of studies on burnout risk (e.g., Maslach and Leiter, 2016), especially the schoolteachers’ risk of burnout (e.g., Pishghadam and Sahebjam, 2012; Saloviita and Pakarinen, 2021), the current study mainly focused on the risk of burnout among Japanese schoolteachers and examined both social and individual factors that potentially influence the reduction of risk. One of the individual factors of Japanese schoolteachers worth noting is the concept of help-seeking preference (HSP). This concept refers to the extent to which individual teachers can seek help from others when they are in trouble (Tamura and Ishikuma, 2001), and has attracted attention in educational and school psychology in Japan. Specifically, Tamura and Ishikuma (2001, 2008) noted that helping each other in the educational workplace could be done after they voluntarily ask their colleagues and managers for help. However, schoolteachers’ tendency to hesitate to ask for help (and to take things on by themselves) may lead to dismal consequences when difficult problems arise. A low level of help-seeking increases the risk of burnout. In the current study, therefore, we regarded HSP as an individual factor of schoolteachers to reduce their risk of burnout.

Furthermore, we surmised that creating a *collegial* organizational climate might play an important role in countering the association between a low level of help-seeking and mental health problems. A collegial climate or the concept of collegiality among schoolteachers can be defined as a relationship in which schoolteachers can share information and exchange their opinions to develop a common understanding, and support and enhance each other in the environment for school education without qualms, regardless of their age or position (Maeda and Hashimoto, 2020; Maeda et al., 2021). This organizational collegial climate in schools has also been a focus in educational and school psychology in the Japanese cultural context. For example, Fuchigami (2005) argued that a collegial climate, wherein schoolteachers can actively exchange opinions and share information, is essential in all educational activities. The organizational climate of schoolteachers has continued to be examined in the field of organizational psychology regardless of cultural contexts, and several studies have examined an important relationship between the organizational climate and schoolteachers’ mental health (e.g., Grayson and Alvarez, 2008). The current study specifically focused on the Japanese cultural context and examined the possibility that creating a collegial climate in school may play an important role in dealing with the various problems faced by Japanese schoolteachers.

In summary, the purpose of the current study was to examine the influence of HSP and the collective perceptions of organizational climate on Japanese schoolteachers’ mental health. Previous studies reported that HSP was negatively associated with the risk of burnout, suggesting that teachers who hesitate to seek help from colleagues are more inclined to have mental health problems (Tamura and Ishikuma, 2006). Based on these findings, the current study tested our hypothesis that a collegial organizational climate would be negatively associated with burnout. We also attempted to develop a new scale that measures teachers’ collective perceptions of the organizational climate in their school. To do so, Study 1 examined the reliability and validity of our newly developed scale, and Study 2 was a test of our hypothesis using the scale we developed in Study 1.

## Materials and Methods

### Study 1: Development of a Scale to Measure Teachers’ Organizational Climate in Schools

Several studies in Japanese educational and school psychology have focused on the organizational climate in schools and have also developed scales to measure the climate (e.g., Fuchigami, 2005; Nishiyama et al., 2009). Existing scales for measuring organizational climate, however, have been slanted to measure the “self-perception” of teachers themselves. Of necessity, the measurement with psychological scales cannot help but rely on the subjectivity of the respondents to the scale, but it is necessary to consider the aspect of the organizational climate of a school as a socio-environmental organizational factor. Therefore, in the current study, we attempted to develop a scale based on the Relational Mobility Scale, which attempts to measure social factors (Yuki et al., 2007). Relational mobility is generally defined as “a socio-ecological variable that represents how much freedom and opportunity a society affords individuals to select and replace interpersonal relationships based on their personal preferences” (Yuki and Schug, 2020). How people living in society collectively perceive their human relations and the social environment in which they live must be measured, rather than simply measuring the human and social relations of each individual. Based on this point, Yuki et al. (2007) have attempted to measure the relational mobility of the people living in a society where the respondents live by using “they” as the subject of the questionnaire items instead of “I.” In the current study, we also recognized the potential of their approach and aimed to develop a scale to assess the socio-environmental organizational climate in schools based on this perspective. Specifically, our newly created scale aimed to measure the perceptions of their colleagues. In Study 1, we first developed a new scale and tested the reliability and validity of the scale. Furthermore, we also attempted to evaluate the test-retest reliability.

#### Participants

Participants were self-selected from a pool of approximately 4.65 million people from a survey agency called Cross Marketing Co.^1^ More specifically, from this pool, we recruited 558 Japanese schoolteachers who worked in elementary, junior high, and high schools (461 men and 97 women with an average age of 52.4 years). The survey agency sent e-mail messages to potential participants and encouraged their participation with monetary incentives. To evaluate the test-retest reliability of our scale, after approximately 1 week, we asked the participants to complete the Teachers’ Perceptions of Organizational Climate Scale a second time. A total of 227 schoolteachers (152 men and 75 women with an average age of 49.9 years) completed it.

#### Measures

Demographic data about gender, age, etc., were collected first. Next, the questionnaire titled “Survey on Education” was presented to the participants with a brief description and information about the study. The following measures were included in the survey questionnaire.

The Teachers’ Perceptions of Organizational Climate Scale was developed for this study. Items on the scale were preceded by the following leading sentence: “We will ask you about the schoolteachers (e.g., coworkers in the schoolteachers’ room) with whom you work at your school. Please answer how much you feel the following statements describe them.” By doing so, we attempted to measure how organizational climate is collectively perceived, and not the perception of the schoolteachers themselves regarding the climate. Furthermore, we developed items to measure the collegial or collaborative organizational climates and closed organizational climates based on previous studies (e.g., Fuchigami, 2005). A sample collegial item included, “There are many coworkers with whom they (the schoolteachers you usually work with) can always mutually discuss and consult with.” An item assessing a closed climate was, “They are mindful of not causing conflicts of opinion.” Response options ranged from “does not describe at all” (1) to “describes very much” (7).

To examine the concurrent validity of our newly developed scale, we utilized the Fuchigami (2005) scale items to measure the “collaborative climate” and “obedience-induced climate.” The response options ranged from “does not describe at all” (1) to “describes very much” (7).

#### Results of Study 1

We first conducted an exploratory factor analysis with Promax rotation. As we predicted, the analysis yielded two factors. We named Factor 1 “Collegial Organizational Climate.” Factor 2 was named “Closed Organizational Climate.” Each factor formed a subscale of the new Teachers’ Perceptions of Organizational Climate Scale. The subscale factor loadings are shown in Table 1.

**TABLE 1:** Factor loadings of the subscales of the teachers’ perceptions of organizational climate scale.

Subscale/item	Factor 1	Factor 2	Communality
**Factor 1: Collegial organizational climate**			
1. They (the schoolteachers you usually work with) foster a social climate where everyone is free to express their opinion, which is acknowledged regardless of their age or position	0.904	−0.011	0.823
2. If there is a problem with the relationship with a student, they are able to mutually point out the problem	0.886	0.004	0.783
3. They often exchange opinions about their educational goals and policies without hesitation	0.885	0.061	0.759
4. They put their heads together so that one teacher would not deal with a student’s problem alone	0.871	−0.027	0.771
5. There are many coworkers with whom they can always mutually discuss and consult with	0.862	−0.032	0.759
6. They often exchange opinions about their educational viewpoints and policies	0.851	0.007	0.721
7. There are many opportunities where they can discuss not only their successes but also failures in teaching	0.847	0.024	0.708
8. There are many opportunities to receive support and advice from colleagues when they have trouble with something	0.825	−0.017	0.688
**Factor 2: Closed organizational climate**			
9. They are mindful of not causing conflicts of opinion	0.083	0.923	0.820
10. They try not to cause discord in their relationships	0.105	0.859	0.703
11. They are cautious of their behaviors so that they are not disliked by their coworkers	0.023	0.827	0.674
12. They often concern themselves with their respective age or positional disparities with their coworkers	0.007	0.737	0.541
13. They often find themselves not being able to do what they want because they are concerned about their relationships with their coworkers	−0.107	0.733	0.589
14. They often turn a blind eye to students’ problems which their coworkers have	−0.269	0.539	0.437

The correlation patterns with the Fuchigami (2005) scale are provided in Table 2. The results showed a significant positive correlation between the Collegial Organizational Climate subscale (Factor 1) and a collaborative climate (*r* = 0.77, *p* < 0.01), and significant positive correlations between the Closed Organizational Climate subscale (Factor 2) and an obedience-induced climate (*r* = 0.61, *p* < 0.01). The Cronbach’s alpha coefficient for the Collegial Organizational Climate subscale was 0.960. The scale measuring school organizational climate used in previous studies (e.g., Nishiyama et al., 2009) has a small number of items. Therefore, our newly created scales have an advantage in this regard. It should be also noted that our attempt to measure the climate of a school organization as a socio-environmental factor demonstrated good concurrent validity and reliability.

**TABLE 2:** Descriptive statistics and zero-order correlation coefficients among related variables in Study 1.

Variables	M (SD)	1	2	3	4	5
**First time period**						
1. Collegial organizational climate	4.53 (1.14)					
2. Closed organizational climate	3.84 (1.00)	−0.27**				
3. Fuchigami (2005) collaborative climate scale	4.52 (1.26)	0.77**	−0.23**			
4. Fuchigami (2005) obedience-induced climate scale	3.84 (0.98)	−0.27**	0.61**	−0.25**		
**Second time period**						
5. Collegial organizational climate	4.44 (1.09)	0.70**	−0.25**	0.67**	−0.27**	
6. Closed organizational climate	3.98 (0.95)	−0.29**	0.41**	−0.22**	0.27**	−0.26**

**p < 0.01.

Furthermore, using the data from second time period, we conducted the confirmatory factor analysis and found that the goodness-of-fit indices were within an acceptable range (CFI = 0.916, RMSEA = 0.118, GFI = 0.824). Correlation analysis was conducted between the scores on the first and second administrations of the scale. The results showed a positive correlation between the two administrations of the Collegial Organizational Climate subscale (*r* = 0.70, *p* < 0.01). The two administrations of the Closed Organizational Climate subscale were only moderately correlated (*r* = 0.41, *p*s < 0.01). The test-retest reliability of the Closed Organizational Climate subscales needs to be further investigated for stability. Given the lower reliability of the measure of closed organizational climate, in Study 2, we examined our main hypothesis focusing only on the Collegial Organizational Climate subscale.

### Study 2: Hypothesis Testing

Our main hypothesis was that a collegial organizational climate would be negatively associated with burnout. To this end, Study 2 examined the influence of HSP and collective perceptions of organizational climate on teachers’ burnout.

#### Participants

We recruited 487 schoolteachers who worked in elementary and junior high schools in Japan (362 men and 125 women with an average age of 49.3 years) through the same survey agency that was used in Study 1 (Cross Marketing Co.).

#### Measures

First, the participants answered items asking for demographic data (gender, age, etc.), then they completed scales assessing organizational climate, HSP, and burnout. We used the Collegial Organizational Climate subscale developed in Study 1 to measure the perceptions of a collegial or collaborative organizational climate. The response options ranged from “does not describe at all” (1) to “describes very much” (7). Cronbach’s alpha coefficient for the Collegial Organizational Climate subscale was 0.960. Schoolteachers’ HSP was assessed based on the study by Tamura and Ishikuma (2001). The items assess the teacher’s desire for and attitudes toward help, and the scale has been validated in a sample of Japanese schoolteachers. Items are responded to using a seven-point Likert scale ranging from “does not describe at all” (1) to “describes very much” (7). To assess burnout, we used the Japanese version revised by Kubo and Tao (1994) of the Maslach Burnout Inventory (Maslach and Jackson, 1981). Although this scale measures three aspects of the burnout syndrome (i.e., depersonalization, physical exhaustion, and personal accomplishment), we focused on depersonalization and utilized the Depersonalization scale (seven items). The Cronbach’s alpha coefficient was 0.93 in the present study.

#### Results of Study 2: Hypothesis Testing

**FIGURE 1:**
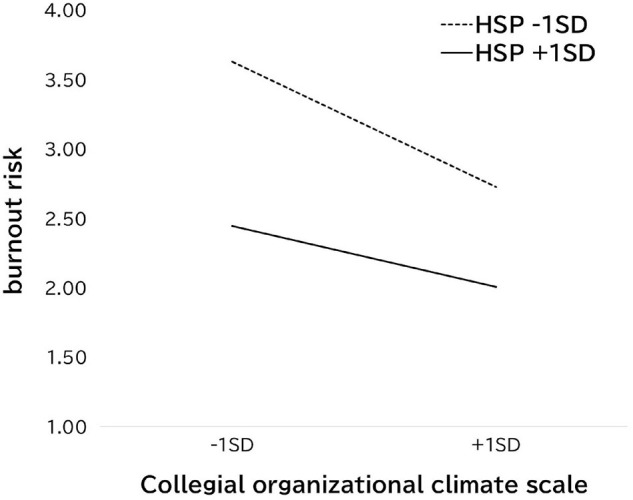
Interaction effect of Japanese schoolteachers’ help-seeking preference and their collective perceptions of collegial organizational climate (dependent variable: burnout risk).

We conducted hierarchical regression analyses to identify predictors of the school teachers’ burnout risk (Table 3). The predictors were gender, age, HSP, collaborative climate, and the interaction of HSP and collaborative climate. The main effects of age, HSP, and collaborative climate were significant predictors. These main effects are understandable: younger schoolteachers with a low level of help-seeking and who perceived the organizational climate as less collegial were at risk of burnout. More importantly, there was a significant interaction effect between the HSP and collaborative climate. The interaction effect indicated that HSP was less closely associated with teachers’ burnout risk if the organization was perceived as having a collaborative climate (Figure 1).

**TABLE 3:** Results of the hierarchical regression analyses predicting schoolteachers’ burnout risk.

Variable	Step 1	Step 2	Step 3	Step 4
Gender	0.088	0.057	0.054	0.063
Age	−0.115*	−0.077	−0.085*	−0.093*
School	−0.066	−0.059	−0.054	−0.048
Personal variable				
HSP		−0.382**	−0.320**	−0.338**
Socio-environmental variables				
Collaborative climate			−0.252**	−0.238**
HSP × Collaborative climate				0.093*
*R* ^2^	0.020*	0.164**	0.224**	0.232**
Δ*R*^2^		0.144**	0.060**	0.008*

*HSP: help-seeking preference. Gender is coded as 0 = female and 1 = male; school is coded as 0 = elementary school and 1 = junior high school; standardized regression coefficients are demonstrated.*

*p < 0.05, **p < 0.01.

## Discussion

In Study 1, we developed the Teachers’ Perceptions of Organizational Climate Scale to assess schoolteachers’ collective perception of the organizational climate in their school and confirmed the reliability and validity of the scale. Factor analysis indicated that the scale had two factors: collegial organizational climate and closed organizational climate. In Study 2, the Collegial Organizational Climate subscale scores were used to predict burnout risk. Furthermore, we examined demographic characteristics and HSP as predictors. The results of Study 2 demonstrated that younger age, a low level of help-seeking, and a less collegial organizational climate were positive predictors of burnout risk. Importantly, a significant interaction effect indicated the importance of creating a collegial organizational climate. Specifically, although a low level of help-seeking was associated with teachers’ burnout risk, this association was alleviated if the schoolteachers perceived their organizational climate to be collegial.

Recently, the realization of “school as a team” and the importance of a collegial organizational climate has come to attract a great amount of attention in Japan. The basic idea of school as a team is that by appropriately sharing roles as a team between the teaching and specialist staff, the teaching staff would be able to focus more on guiding students, such as in the form of lessons. In this sense, the main purpose of the school as a team is to improve the educational function of schools. We accept this idea but also consider that it would help in tackling schoolteachers’ mental health problems, such as the risk of burnout. As our results suggest, the concept of school as a team might help schoolteachers not only to increase their focus on guiding students, such as in the form of lessons but also have a preventive effect on the development of mental health problems. In other words, the importance of team support, which means to effectively support students as a whole by maximizing the use of human resources (managers, colleagues, *Yogo* teachers, school counselors, school social workers, parents, etc.) in the school organization and coordinating them, is primarily for the benefit of the students, but it is also important for the teachers themselves. In this sense, the school as a team concept can be realized as one potential way to create a collegial organizational climate that is beneficial to both students and schoolteachers and, at the same time, helps enhance the help-seeking of individual schoolteachers.

We believe that there are some implications of the current study, in which we conducted a survey of Japanese schoolteachers, developed a new scale measuring their collective perceptions of organizational climate, and highlighted the importance of creating a collegial climate as a socio-environmental factor. Particularly, if we take the nature of the Japanese society being collectivistic into account, we should consider the possibility that it is more difficult to create a collegial climate in a collectivist society as a result of the adaptive nature of being concerned other’s view. Over the last 25 years, the construct of individualism and collectivism (Hofstede, 1980; Triandis, 1995; see also Triandis and Gelfand, 1998) and related construct of self-construal (Markus and Kitayama, 1991) have garnered the attention of researchers in psychology and culture. Based on a framework for understanding these cultural differences from a sociological perspective (Hashimoto and Yamagishi, 2013, 2016; Yamagishi and Hashimoto, 2016), culturally shared beliefs and culture-specific cognitions and behaviors are viewed as tools for adapting to collectively constructed social niches. From this perspective, it is possible that due to the way Japanese schoolteachers behave to avoid being disliked by others and be perceived as someone who interferes unnecessarily, they do not ask for or offer assistance to others, which hinders creating a collegial climate. The question of how to create a collegial culture in an organizational setting is an important issue for future studies, given that the Japanese mentality is prone to dissociation between ideals and reality because they are concerned about what others think, even if they want to act according to their ideas (see Hashimoto and Yamagishi, 2015; Hashimoto, 2021).

Although the current study yields important insights, several limitations need to be addressed. First, it should be considered to more carefully examine the cultural-universality and/or cultural-specificity regarding the potential determinants that alleviate schoolteachers’ risk for burnout; based on substantial previous research on cultural, regional, and age differences in the level and causes of burnout (for review, Sharma and Cooper, 2016), it is necessary to elaborate the findings not only through surveys of Japanese schoolteachers but also through cross-cultural surveys in the future. We should also acknowledge the fact that our sample might be biased in that we sampled only those who had access to our web-based surveys, which is a potential limitation that needs to be addressed through future research. Additionally, the Teachers’ Perceptions of Organizational Climate Scale should be considered a preliminary scale that acts as a measure of the collective perceptions of organizational climate. However, a more detailed study of its validity and psychometric properties will be necessary for the future; our scale items are also a bit too formal and could be modified in the future. Nevertheless, because mental health problems among in-service teachers in Japan are an urgent issue, the field must place greater importance on promoting a collegial organizational climate and evaluating its effects, as suggested by this study’s findings. As a future direction, we should attempt to link the current findings to concrete proposals on how to create a collegial organizational culture, taking into account the difficulties of the collectivist Japanese society.

## Data Availability Statement

The raw data supporting the conclusions of this article will be made available by the authors, without undue reservation.

## Ethics Statement

The studies involving human participants were reviewed and approved by the Yasuda Women’s University. Written informed consent was not provided because the survey agency, Cross Marketing Co., (http://global.crossm.co.jp), sent e-mail messages to potential participants, and encouraged their participation with monetary incentives. Those who did not agree to participate were unable to do so. Therefore we considered that all participants agreed to participate in our survey.

## Author Contributions

HH and KM contributed to the study design. HH wrote the entire manuscript. Both authors collected and analyzed the data, contributed to the article, and approved the submitted version.

## Conflict of Interest

The authors declare that the research was conducted in the absence of any commercial or financial relationships that could be construed as a potential conflict of interest.

## Publisher’s Note

All claims expressed in this article are solely those of the authors and do not necessarily represent those of their affiliated organizations, or those of the publisher, the editors and the reviewers. Any product that may be evaluated in this article, or claim that may be made by its manufacturer, is not guaranteed or endorsed by the publisher.

## Footnotes

1
https://www.cross-m.co.jp/en/
